# A head and neck cancer intervention for use in survivorship clinics: a protocol for a feasibility study

**DOI:** 10.1186/s40814-016-0061-3

**Published:** 2016-05-05

**Authors:** Talya Salz, Mary S. McCabe, Kevin C. Oeffinger, Stacie Corcoran, Andrew J. Vickers, Andrew L. Salner, Ellen Dornelas, Rebecca Schnall, Nirupa J. Raghunathan, Elizabeth Fortier, Shrujal S. Baxi

**Affiliations:** 1 Memorial Sloan Kettering Cancer Center, 1275 York Ave, New York, NY 10021 USA; 2 Hartford Hospital, 80 Seymour St, Hartford, CT 06102 USA; 3 Columbia University School of Nursing, 617 W 168th St, New York, NY 10032 USA

**Keywords:** Head and neck cancer, Survivorship, Patient-reported outcomes

## Abstract

**Background:**

Head and neck cancer survivors commonly experience severe long-term toxicities, late-occurring symptoms, and significant risks of the second primary malignancy and comorbid illnesses. With multiple simultaneous health issues, these complex cancer survivors often do not receive comprehensive health care that addresses their needs. A tool is needed to streamline and standardize comprehensive care for this cohort.

**Methods/design:**

We designed the Head and Neck Survivorship Tool: Assessment and Recommendations (HN-STAR) to address health care challenges for head and neck cancer survivors. HN-STAR is an electronic platform that aims to simplify the provision of personalized care in cancer survivorship clinics. It uses an algorithmic approach to integrate patient-reported outcomes, clinical details, and evidence-based guidelines to standardize comprehensive care provided in routine survivorship visits. It has four integrated components: (1) a simplified *treatment summary*, which pulls treatment details from a clinical database or can be completed manually using a streamlined form; (2) an online *self-assessment* for patients to report their own symptoms; (3) an interactive *discussion guide* presenting all relevant information to the provider during the clinic visit; and (4) a *survivorship care plan* generated at the end of each visit that reflects decisions made during the visit. By using a modifiable electronic platform, HN-STAR provides a method for incorporating survivorship care plans into clinical practice and for disseminating evidence on symptom management and preventive care.

This is a study to assess the feasibility of a future multi-site, randomized clinical trial of HN-STAR. We will enroll head and neck cancer survivors who are followed in one of two nurse practitioner-led survivorship clinics. We will implement HN-STAR for one routine survivorship visits. We will assess (1) usability and feasibility outcomes of HN-STAR from the perspective of key stakeholders and (2) the planned outcomes intended for the larger trial. We will collect usability and feasibility data from online surveys of survivors and their providers. Our findings will inform whether it is feasible to advance HN-STAR to trial. If so, we will adapt HN-STAR and the study design of the trial in response to feedback from survivors and providers. The long-term goal is to determine if such an intervention will lead to improved and simplified comprehensive survivorship care.

**Discussion:**

This feasibility study will evaluate implementation of HN-STAR into clinical practice in terms of usability, practicality, and clinical flow in two distinct clinical settings. This study will also provide critical baseline data to characterize this vulnerable population. Findings from this study will inform a multicenter randomized trial of HN-STAR, aimed at standardizing and streamlining the delivery of evidence-guided comprehensive care for head and neck cancer survivors. Ultimately, if found effective, the modular structure of HN-STAR could permit its expansion to survivors of other complex cancers.

**Trial registration:**

ClinicalTrials.gov, NCT02571673

## Background

After cancer treatment is complete, cancer survivors need a new approach to their ongoing care. Comprehensive survivorship care involves routine surveillance for recurrence and new cancers, detection and management of chronic and late-developing toxicity (together called “late effects”), and management of comorbid conditions. For some survivors, the risks of recurrence and late effects are low, and a primary care provider can effectively oversee comprehensive survivorship care with minimal involvement of oncology providers. Other groups of cancer survivors, however, have more complex needs and require continued follow-up with their oncology providers [1].

Head and neck cancer patients are one such group of complex cancer survivors who confront numerous and serious health challenges beyond the risk of local recurrence. Advances in treatment, specifically concurrent radiation and chemotherapy, have improved survival in head and neck cancer but have also led to an increase in chronic and late-developing toxicity (late effects) [2–7]. Some common late effects include hearing loss, dry mouth, decreased taste, neck fibrosis, and lymphedema in the neck and face [8–11]. More debilitating late effects include destruction of the jaw, inability to speak, difficulty swallowing, and difficulty opening the mouth [12–16]. Up to half of head and neck cancer survivors are diagnosed with psychological distress [17–19].

Because many head and neck cancers arise in the setting of chronic tobacco or alcohol exposure, these patients also often have other tobacco-related comorbid illnesses, such as other cancers, pulmonary disease, and cardiovascular disease, and can have multiple non-cancer health care providers [20–26].

With such complex needs, comprehensive survivorship care may be difficult to deliver. The central focus of survivorship care in head and neck cancer is early identification of recurrent and second head and neck cancers by an oncology provider [1]. Although surveillance by oncology providers also includes the identification and management of late effects, there is no central clearinghouse for guidelines or standards in head and neck cancer, suggesting that methods for addressing late effects likely vary by provider or by clinical practice.

Beyond oncologic surveillance, management of non-oncologic care is necessary to improve survival. Primary care should include aggressive management of comorbid illnesses, risk modification (e.g., tobacco cessation), completion of recommended cancer screening for new (non-head and neck) cancers, vaccination, and receipt of general preventive care [21, 27]. Unfortunately, in two studies, between 18 and 50 % of head and neck cancer survivors reported ever seeing a primary care provider [28, 29]. The failure to receive primary care has been documented among survivors of other cancers [30–32]. In turn, cancer survivors who do not receive primary care are less likely to receive preventive services and appropriate interventions for comorbidities than those who do [30, 31, 33–36].

Addressing multiple medical issues simultaneously, and identifying which provider is responsible for management, can complicate a cancer survivor’s ongoing care. In its landmark report, *From Cancer Patient to Cancer Survivor: Lost in Transition*, the Institute of Medicine (IOM) recommended the use of survivorship care plans to facilitate coordination of survivorship care between oncology and primary care providers [37]. A survivorship care plan is a document given to the patient by oncology providers at the end of treatment that includes (1) a treatment summary and (2) a plan of care describing late effects and recommendations for interventions and self-management [37]. The survivorship care plan, which is shared with the primary care provider, includes explicit plans for who is responsible for each aspect of care. Survivorship experts have widely endorsed the use of survivorship care plans, and multiple professional societies have encouraged their use [38–45]. However, the development and consistent implementation of survivorship care plans in clinical practice have been challenging [46–52]. The major barriers to the use of survivorship care plans are the time and personnel required to create them and the difficulty reviewing their content during routine visits [46–50, 52]. These barriers may be particularly problematic for complex cancer survivors, like head and neck cancer survivors, who may have multimodality treatment histories, have treatment-based surveillance recommendations, experience numerous persistent toxicities, be at risk for late effects, require management of comorbidities, and need modification of multiple risk factors—all of which should to be noted in survivorship care plans.

**Fig. 1:**
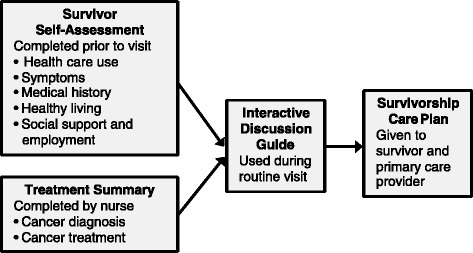
HN-STAR components and clinical flow

We developed a web-based, algorithm-driven platform called the Head and Neck Survivorship Tool: Assessment and Recommendations (HN-STAR) to address the most salient issues in providing comprehensive survivorship care to head and neck cancer survivors. First, HN-STAR ensures the identification of all late effects by collecting symptom data directly from patients. It then synthesizes patient-reported outcomes, treatment data, and current evidence about survivorship care into a tailored interactive discussion guide. The oncology provider uses the interactive discussion guide in a routine oncology follow-up visit to address all elements of comprehensive survivorship care. Finally, HN-STAR automatically creates the survivorship care plan based on the clinic visit, which minimizes burden for oncology providers. This survivorship care plan can be updated at each visit to incorporate symptom changes, modified management plans, and more current evidence regarding survivorship care (Fig. 1).

The goal of our protocol is to evaluate the feasibility of HN-STAR in the setting of survivorship clinics, in preparation for a future multi-site randomized controlled trial. The future trial will randomize clinics at multiple centers to use HN-STAR or usual care, and the primary trial outcomes will be changes in (1) the number of late effects identified and (2) the number of late effects managed in clinic. We will also investigate adherence to recommended care and any changes in health outcomes.

The current protocol is a feasibility study to inform preliminary outcomes, design, and sample size for the future trial. Specifically, we are interested in usability of our interface for patients and providers, feasibility of study conduct (recruitment and completion), feasibility of data collection using a web interface and medical record abstraction, clinical and demographic features of our study sample, and differences in our primary outcomes before and after the intervention. These outcomes will guide whether to advance HN-STAR to a randomized clinical trial of its effectiveness. If HN-STAR will advance to trial, we will use our findings to adapt HN-STAR and the design of the trial.

## Methods/design

### Overview

The purpose of this study is to evaluate the feasibility of HN-STAR as an intervention to streamline comprehensive survivorship care for head and neck cancer survivors in a randomized, multi-site clinical trial setting. We will incorporate HN-STAR into a routine survivorship clinic visit, in order to assess the use of HN-STAR in clinical practice. Prior to a routine clinic visit, each patient will report his or her medical history, preventive care, and symptoms online using the survivor self-assessment. This information (as well as other medical history information) will populate an interactive discussion guide for the nurse practitioner (NP) to use during the clinic visit. Decisions regarding management will be recorded in HN-STAR to generate the survivorship care plan. Each patient and his or her primary care provider (PCP) will receive the automatically generated survivorship care plan. We will assess feasibility outcomes from the patient, his or her NP, and his or her primary care provider. We will also abstract key information from the medical record on clinical outcomes.

### Eligibility and recruitment

#### Head and neck cancer survivors

Eligible patients must be scheduled to receive routine follow-up in the survivorship clinics at Memorial Sloan Kettering Cancer Center (MSK) and Hartford Hospital (HH). In order to be seen at the clinics, they must (1) have completed treatment for head and neck cancer at least 1 year prior to survivorship visit, (2) have no evidence of disease, (3) have a primary care provider on record, and (4) be at least 18 years old. To be eligible for the study, they must be able to provide informed consent and be able to speak and read English. Patients with cognitive, visual, or motor impairment such that they cannot complete the survivor self-assessment (as assessed by the research team) will be excluded.

Two weeks before each patient’s scheduled survivorship clinic visit at either MSK or HH, eligible head and neck cancer survivors will be invited by mail to participate in the HN-STAR study. Patients will provide informed consent over the phone with the research assistant, in accordance with MSK IRB #15-245 (Clinicaltrials.gov number: NCT02571673). The patient will complete the survivor self-assessment on a laptop or tablet before the clinic visit (at home or in the waiting room).

#### Primary care providers

After a participant has attended the survivorship clinic visit and received the survivorship care plan, their identified PCP will be mailed the survivorship care plan. One week later, at each site, the research assistant will contact the PCP associated with the participant by mail and follow up by telephone to invite them to complete an online survey. Through the consent process, participants will be informed that their PCP will be surveyed and interviewed about HN-STAR as part of the study.

#### Nurse practitioners

We will ask the two NPs to provide feedback (via surveys and interview) about their experiences using HN-STAR. Through the consent process, participants will understand that their NP will be surveyed and interviewed about HN-STAR as part of the study.

### Usual care setting

We will implement HN-STAR in routine follow-up visits in two survivorship clinics—the Head and Neck Survivorship Clinic at MSK and the clinic of Gray Cancer Center’s Survivorship Program at HH. The two separate sites will allow us to investigate HN-STAR with the electronic access of claim data (MSK) and without (HH), to inform the scalability of HN-STAR. Each clinic is led by a single NP. The head and neck survivors seen in each clinic have completed treatment for head and neck cancer at least 1 year prior and have no evidence of disease. In standard care, the NP provides oncologic follow-up, creates and delivers a survivorship care plan, addresses healthy behaviors, and ensures that the survivor has a primary care provider who will manage general preventive care. At MSK, the NP continues to follow the survivor annually; at HH, the NP provides a one-time consultative visit before the survivor continues to visit their oncologist.

### Intervention

HN-STAR has four components, described below.

#### Survivor self-assessment

The HN-STAR *survivor self-assessment* includes validated items whenever possible. First, the survivor self-assessment elicits the presence and burden of toxicities using relevant items from the National Cancer Institute’s Patient-Reported Outcomes version of the Common Terminology Criteria for Adverse Events (PRO-CTCAE) [53]. For symptoms that are specific to head and neck cancer but not included in existing PRO-CTCAE measures, we have created items using the same format and symptom attributes as existing items. These symptoms are bleeding from the mouth, trismus, hearing loss, jaw pain, neck or shoulder stiffness, neck pain, pain with swallowing, and bad breath. In addition, the survivor self-assessment includes items regarding medical history and preventive health. Items from validated screening instruments are used to identify alcoholism, tobacco use, physical activity, sexual function, and depression, as shown in Table 1 [54–58]. For other health behaviors, ad hoc assessments are based upon guidelines and institutional consensus at MSK and HH [59–66]. Patients fill out the survivor self-assessment online before the visit, either at home or in the clinic waiting room.

**Table 1:** Standardized items in survivor self-assessment

Construct	Source
Symptom: memory, insomnia, fatigue, tiredness, or lack of energy, numbness or tingling in your hands or feet, shortness of breath, cough, ringing in your ears, dry mouth, voice changes, nosebleeds, mouth or throat sores, pain (general), difficulty swallowing, dizziness	Patient Reported Outcomes—Common Terminology Criteria for Adverse Events [53]
Symptom: difficulty hearing, neck or shoulder stiffness, neck pain, jaw pain, pain in your mouth, pain in your throat, frequency of pain (general), difficulty with opening your mouth, bad breath, bleeding from your mouth	Based on Patient Reported Outcomes—Common Terminology Criteria for Adverse Event
Symptom: sexual function	The European Organization for Research and Treatment of Cancer Quality of Life Questionnaire-C30—Head and Neck-35 [95]
Symptom: depression	Patient Health Questionnaire-2 [55]
Physical activity: frequency	Godin Leisure-Time Exercise Questionnaire [56]
Physical activity: average exercise time	Adaptation of the Godin Leisure-Time Exercise Questionnaire [56]
Smoking status: at least 100 cigarettes in entire life	National Health Interview Survey^a^
Smoking status to determine need for smoking cessation (ever smoked cigarettes, smoking in the past 30 days)	National Comprehensive Cancer Network Smoking Cessation Guidelines^b^
Smoking status: years as a smoker cigarette exposure to determine eligibility for lung cancer screening (cigarettes per day, time since quitting)	Lung Cancer Screening Decision Tool^c^
Smoking history and current status to determine need for smoking cessation (ever smoked cigarettes, smoking in the past 30 days)	National Comprehensive Cancer Network Smoking Cessation Guidelines^b^
Alcohol alcoholism use	The CAGE questionnaire [54]

^a^National Health Interview Survey, Questionnaires, Datasets, and Related Documentation 1997 to the present, centers for disease control and prevention

^b^NCCN CLinical Practice Guidelines in Oncology, Smoking Cessation, Version 1.2015, 2015

^c^Lung Cancer Screening Decision Tool, mskcc.org Prediction Tools, Memorial Sloan Kettering Cancer Center, 2014

#### Treatment checklist

The *treatment checklist* uses claim data from the head and neck cancer diagnosis and treatment. The technical terms will then be translated into lay language. This will be done differently at the two participating sites.

1. At MSK, we will incorporate the electronic claim data into our platform. HN-STAR generates an Automated Treatment Checklist, which presents an organized list of diagnosis, staging, and treatment received at MSK using claim data from billing codes in the MSK record. The NP is prompted to verify the accuracy of the presented list and make necessary corrections.
2. At HH, where claim data will not be automatically ported to HN-STAR, there is a Manual Treatment Checklist, in which all possible diagnosis and treatment options are presented as an organized checklist. The NP must manually complete the Manual Treatment Checklist by referring to the patients’ medical records.

At either institution, once a patient has agreed to participate in the study, prior to the participant’s survivorship clinic visit, the NP will verify or complete the checklist. The checklist will result in the generation of a lay language treatment summary that will appear in the survivorship care plan (described below). The NPs at both sites will be trained on how to use the treatment checklist.

#### Interactive discussion guide

**Fig. 2:**
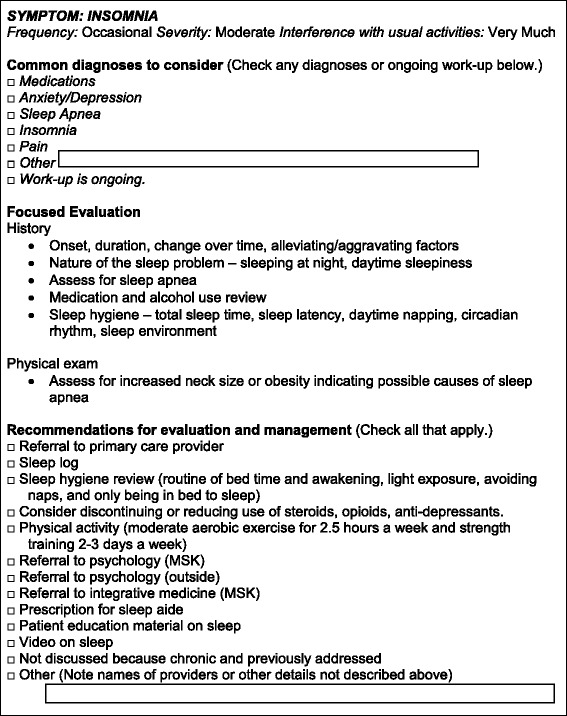
Excerpt of interactive discussion guide for insomnia

The *interactive discussion guide* integrates responses from the survivor self-assessment, verified data from the treatment checklist, and an evidence base for survivorship care. Three algorithms will then use these data to generate the interactive discussion guide that the NP can use during the routine visit (Fig. 2).

1. *Treatment algorithms* use Current Procedural Terminology (CPT) codes and National Comprehensive Cancer Network (NCCN) guidelines to generate personalized surveillance recommendations (e.g., annual thyroid studies for survivors who received radiation to neck) [1].
2. *Symptom algorithms* use PRO-CTCAE responses to identify toxicities of treatment and other relevant issues to address. In the interactive discussion guide, seen only by the NP, these symptoms populate an evidence-based list of common diagnosis to consider, recommendations for focused evaluation, and recommended management options. We have developed these recommendations based on existing evidence when available and institutional consensus within MSK and approval from HH otherwise. We will refine these guidelines as new evidence emerges [40, 67–78].
3. *Prevention algorithms* use patient responses and demographic information to generate a list of personalized prevention recommendations, adapted from the US Preventive Service Task Force and NCCN survivorship guidelines [1, 59–66, 79–82]. Using the interactive discussion guide, the NP will discuss ongoing care and select management plans with the survivor. Selected symptom management plans are entered into HN-STAR and populate the survivorship care plan (described below).

The interactive discussion guide is intended only for the NP to use during the clinic visit. The NP at each site will be trained on how to use the interactive discussion guide.

#### Survivorship care plan

Finally, the *survivorship care plan* will present a treatment summary and plan of care. HN-STAR generates a survivorship care plan after each visit. The treatment summary contains a plain-language cancer history. The plan of care contains personalized recommendations for cancer surveillance, management of late effects, and preventive care, reflecting discussions and decisions from the clinic visit. It also reports a list of non-cancer conditions reported by the patient. Each recommendation includes a schedule and clear delineation of who is responsible. The plan of care also contains generic survivorship information, with a description of signs and symptoms to report to the oncology provider, contact information for the oncology provider, and recommendations to visit a primary care provider.

### Clinical flow using HN-STAR

We will test the feasibility of using HN-STAR over the course of a single, routine survivorship visit at either MSK or HH.

*Before the survivorship visit*: At MSK, the institutional database of CPT codes will automatically populate the Automated Treatment Checklist in HN-STAR, and the NP will verify its accuracy against the medical record. At HH, the NP will use the medical record to complete the Manual Treatment Checklist. At both sites, the treatment checklist will inform treatment-based recommendations in the interactive discussion guide and generate a plain-language treatment summary for the survivorship care plan.

*Before the survivorship visit*: Survivors will complete the online survivor self-assessment. Using the survivor self-assessment and treatment checklist data, HN-STAR will create the interactive discussion guide for the clinic visit that presents (1) an oncologic surveillance schedule, (2) a list of severity-based symptom management options, and (3) personalized preventive care and screening recommendations.

*During the visit*: The NP will use the interactive discussion guide to facilitate conversation. Specifically, the NP will conduct a physical exam, and the NP and survivor will discuss ongoing care, select plans for symptom management, and identify who is responsible for each action (NP, survivor, or primary care provider).

*At the end of the visit*: The treatment checklist will be combined with the selected plan of care from the visit to create a survivorship care plan. At the end of the visit, the survivorship care plan will be printed, given to the survivor and discussed with the survivor. The research assistant will also offer to send a printer-friendly version of the survivorship care plan to the survivor, if the survivor wishes to provide an email address. After the visit, a printed version of the survivorship care plan will be sent to each survivor’s primary care provider.

### Study end points

This study uses surveys at multiple time points to elicit feedback from patients, NPs, and PCPs regarding the use of this system. In addition, to inform whether trial outcomes can be feasibly collected, and to inform a pretest-posttest analysis, we will collect from HN-STAR and the medical record indicators of care received, to establish baseline measures and inform feasibility of collecting data for a future trial. A subset of these indicators will be collected from medical records before and after the clinic visit, using a pretest-posttest design. These end points are described in more detail in the following sections.

#### Study end points from surveys

Surveys will collect data from each survivor, his or her NP, and his or her PCP. All surveys are self-administered in HN-STAR, and responses are recorded and stored electronically. Survey end points are described below and in Table 2.

**Table 2:** Study end points

Assessment	Source	Timing	End points
Survey metrics			
Survivor Post-Assessment Survey	Patient	Upon completion of survivor self-assessment, before the clinic visit	• Perceptions of information quality, system quality, and usefulness, ease of use
Survivor Post-Visit Survey	Patient	Following clinic visit	• Opinion on the usefulness of the survivor self-assessment in the clinic visit • Ease of use of, satisfaction with, and perceived usefulness of the survivorship care plan
Primary Care Provider Survey	PCP	One week after being mailed survivorship care plan, within a month of enrollment into study	• Whether they received and reviewed the survivorship care plan • Ease of use of, satisfaction with, and perceived usefulness of the survivorship care plan
Nurse Practitioner Post-Visit Survey	NP	Following each clinic visit	• Whether the interactive discussion guide presented a complete list of issues for the patient • Whether it contained irrelevant information • Length of the visit
Nurse Practitioner Interview	NP	After all patients have completed clinic visits	• Problems with and benefits of interactive discussion guide • Whether the usefulness or usability of the interactive discussion guide varied by type of patient seen in clinic
Non-survey metrics: HN-STAR			
Automated Treatment Checklist	MSK NP	Immediate concurrent data collection before study visit	• Accuracy of automatic treatment summary generation • Time required to verify
Manual Treatment Checklist	HH NP	Immediate concurrent data collection before study visit	• Time required to complete checklist
Survivor self-assessment	Patient	Immediate concurrent data collection before study visit	• The amount of time taken • The items skipped • The proportion of the self-assessment completed • Receipt of routine preventive care (including cancer screening tobacco cessation, immunizations, and routine general testing)
Non-survey metrics: clinic note			
	NP	Part of routine care, abstracted from any time in the year before the visit (pretest) and in the note pertaining to the clinic visit (posttest)	• Late effects identified and addressed • Receipt of head and neck surveillance • Receipt of appropriate follow-up (e.g., smoking cessation, dental exam, blood work for thyroid studies, endoscopic exam, and head and neck physical exam)

1. *Survivor Post-Assessment Survey*: After completing the survivor self-assessment online, each survivor will complete the Survivor Post-Assessment Survey regarding perceptions of the survivor self-assessment [83, 84].
2. *Survivor Post-Visit Survey*: When the patient comes for the clinic visit, the research assistant will be at the clinic and available to answer questions about the study and direct the integration of study flow in clinical practice as needed. Each patient will see the NP (who will use the interactive discussion guide during the visit) and receive the survivorship care plan. The survivor will then complete the Survivor Post-Visit Survey on a computer or iPad. The Survivor Post-Visit Survey elicits participant opinion on the clinic visit and the survivorship care plan [83–86].
3. *Primary Care Provider Survey*: Two weeks after the visit (when the survivorship care plan is mailed to the PCP), each patients’ PCP will be invited to complete a brief online survey regarding the survivorship care plan [87–89].
4. *Nurse Practitioner Post-Visit Survey*: Directly after each clinic visit, the NP will complete a brief online survey regarding the experience of using the interactive discussion guide during the visit [88–90].
5. *Nurse Practitioner Interview*: After all survivors have completed feasibility testing, the research assistant will conduct a qualitative interview with each NP to evaluate the use of the interactive discussion guide and creation of the survivorship care plan.

#### Study end points from HN-STAR and medical records

Survivor and nurse practitioner data will be recorded through the web interfaces of HN-STAR. In addition, NPs will document clinic notes as part of routine care, as described below and in Table 2.

1. For all visits at MSK, preceding the patient visit, the NP will verify the Automated Treatment Checklist. When the NP makes any changes to imported data and verifies the final treatment checklist, these changes will be recorded and will inform the accuracy of the automatically generated treatment summary and the time required to verify the information.
2. For all visits at HH, preceding the patient visit, the NP will complete the Manual Treatment Checklist. Data will be collected regarding the time required to complete the checklist.
3. All patients will complete the survivor self-assessment before their clinic visit. HN-STAR will record the amount of time taken, the items skipped, and the proportion of the self-assessment completed. In addition, the survivor self-assessment records the receipt of non-oncologic care, such as screening for new cancers, vaccination, and other preventive care elements that are explicitly collected in the survivor self-assessment.
4. As part of routine care, the NP will record topics addressed and actions taken for each visit (including referrals, prescriptions, and other management plans) in the clinic note as part of routine care. These cancer-related, or oncologic, outcomes indicate care received, and they include late effects (symptoms) identified and addressed, receipt of head and neck surveillance, and appropriate follow-up (e.g., dental exam for those who underwent radiation therapy and tobacco cessation referral for those who smoke). Our primary oncologic outcomes are the identification and management of late effects. For each late effect assessed in HN-STAR, we will consider it identified if it was mentioned in a clinic note, and we will consider it addressed if there was a referral, recommendation, education, or explicit acknowledgement of inaction. (Inaction may be appropriate, because in some cases, when symptoms are persistent and intractable, no intervention may be recommended.) Clinic note data will be used as part of a pretest-posttest design, described in further detail below. The research assistant at each site will retrieve data regarding late effects identified and addressed in the year preceding the study visit and during the study visit, to assess changes before and after the HN-STAR intervention. The research assistant will also assess oncologic surveillance occurring in the year preceding the study visit and during the study visit from the medical record, using a medical record data abstraction form.

### Sample size

This feasibility study is not powered to test formal hypotheses, such as differences in end points by patient characteristics. Instead, it will provide feedback regarding, and outcomes from, the HN-STAR process. We will recruit 45 patients: 30 from MSK and 15 from HH. This sample size is likely adequate to reach thematic saturation. We estimate that this sample size is feasible within the two clinics and should provide variability between survivors on factors such as age, gender, diagnosis, comorbidities, risk factors, and competency with computers. Further, a sample size between 24 and 50 is recommended for pilot studies to estimate sample sizes for a future trial [91–93].

We will continue to enroll patients until 45 patients reach the end of the survivor self-assessment. Patients who skip items in the survivor self-assessment, who skip items in the surveys, or who terminate a study visit early will be included in the feasibility analysis, as these actions provide important feasibility data. However, any patients who do not reach the last web page of the survivor self-assessment will be excluded from the study, because the patient needs to reach the final web page of the self-assessment (even if all items within it are skipped) in order to create the interactive discussion guide.

### Analysis of feasibility metrics

The structured survey questions will be summarized using descriptive statistics. We will calculate means and standard deviations for continuous variables and counts and percentages for categorical variables, presented with 95 % confidence intervals. Quantitative outcomes will include survivors’ rates of completion of the self-assessment and survey completion rates for survivor, NP, and Primary Care Provider Surveys [94]. The number of questions to answer is different between surveys and between respondents, based on skip patterns built into the electronic survey platform. Each survey will be considered complete if at least 75 % of questions that are asked are answered. Other quantitative outcomes include survivors’ participation rate (including clinical and demographic descriptors of participants and non-participants as well as reasons for non-participation), when the survivor self-assessment was completed (indicating whether it was at home or just prior to the visit), the median time survivors required to complete the self-assessment, the accuracy of the automated treatment summary (verified against the EMR), the time required for NP verification of the treatment summary, and the length of HN-STAR visits.

Each component of HN-STAR will be evaluated individually for feasibility. We will consider the survivor self-assessment feasible for subsequent effectiveness testing if the following benchmarks are met:

- >75 % survivors completed >75 % of the items within the self-assessment.
- The mean proportion of assessment completed >75 %.
- The median time to complete self-assessment <15 min.
- >50 % of survivors rate the self-assessment visit positively on the Survivor Post Assessment Survey.

We will consider the Automated Treatment Checklist feasible if the following benchmarks are met:

- >90 % of the treatment summaries were deemed accurate.
- The median time to verify the summary <20 min.

We will consider the Manual Treatment Checklist feasible if the following benchmarks are met:

- The median time to complete <30 min. (In practice, survivorship care plans typically take an hour or more to complete [47]).

The interactive discussion guide will be considered feasible if the following benchmarks are met:

- >75 % of interactive discussion guides did not miss relevant information. We will determine whether relevant information was covered using the Nurse Practitioner Survey.
- >50 % of survivors rate the survivorship visit positively on the Survivor Post Assessment Survey.
- The median visit time <50 min (current visit time average is 40 min).

The survivorship care plan will be deemed feasible if the following criteria are met:

- >50 % of survivors rate the survivorship care plan positively, as rated in the Survivor Post-Visit Survey.
- >50 % of primary care providers rate the survivorship care plan positively in the Primary Care Provider Survey.

If any component does not meet all criteria, we will consider further adapting the component as needed and testing them further in a future protocol.

### Analysis of preliminary metrics of processes of care

We will use descriptive statistics to report receipt of oncologic and non-oncologic care, as described below.

#### Oncologic outcomes

For receipt of appropriate cancer-related surveillance, we will use descriptive statistics to report each element of oncologic care that each patient received. Each patient may not require every element of follow-up, depending on their primary tumor site or treatment received. These cancer-related elements of care may include a history and physical, thyroid exam, recommendation to receive a dental exam, and referral to tobacco cessation. We will use the number of recommended elements of care (based on survivor characteristics) as the denominator, and we will calculate the proportion of these elements that are performed as indicated in the clinic note. To quantify the management of late effects, for each patient, we will first count the number of late effects identified in the survivor self-assessment. We will then calculate the proportion of identified late effects that are addressed.

We will calculate the difference in each oncologic outcome before HN-STAR use and after. Differences in oncologic metrics will be used in the power calculation for the proposed randomized trial. Any observed differences may in part be due to performance bias, with the NP being more attentive during a study visit, and will be interpreted cautiously.

#### Non-oncologic outcomes

The non-oncologic outcomes (e.g., cancer screening and vaccination) in HN-STAR are collected only once in the survivor self-assessment, before the clinic visit. These metrics will be reported descriptively to provide baseline measures and inform the feasibility of collecting these data from self-report. Only some of these elements will be recommended for each patient, depending on the patient’s age, sex, and behaviors. We will determine the number of recommended elements for each patient and calculate the proportion of recommended elements of non-oncologic care that are performed per patient prior to the clinic visit. In addition, for each element of non-oncologic care, we will determine the number of patients for whom that element is recommended and calculate the proportion of patients who received it as recommended prior to the clinic visit.

### Adaptation and advance of HN-STAR

If HN-STAR is deemed feasible for further effectiveness testing, using the benchmarks described above, we will use our findings to adapt the content of HN-STAR. Specifically, the recruitment rate will guide estimates of timelines for clinical recruitment. Skipped items on the survivor self-assessment and surveys will guide the revision and adaptation of these instruments. If needed, we will consider assessing often-missing data elements from other sources. Other feasibility data may inform how to integrate HN-STAR into the clinical flow more effectively or how to ease data collection for patients.

The primary outcomes of the future trial will be the identification and management of a selection of the late effects measured in HN-STAR. We will base the selection of late effects on prevalence, demonstration of unmet needs, changes in how often a late effect is addressed between pretest and posttest, and feasibility of measurement and data collection.

We will also use the baseline rates and pretest-posttest changes in rates of the identification and management of late effects to estimate the sample size for the future trial.

We will then advance HN-STAR to a randomized clinical trial to assess its effectiveness in improving the health outcomes assessed in aim 2.

## Discussion

This feasibility study will evaluate implementation of HN-STAR into clinical practice in terms of usability, practicality, and clinical flow. HN-STAR requires multiple changes to usual care, and we will use stakeholder feedback to ease the implementation of HN-STAR in a trial. Survivors will report how comfortable they are with reporting their own symptoms online, having the nurse practitioner use a computer in clinic to guide their care, and receiving a survivorship care plan. For example, based on patient feedback, we may improve the interface of assessing symptoms or change the content or layout of the survivorship care plan. NPs will report their own comfort with creating a treatment summary and using a computer during the clinical encounter and automatically generating a survivorship care plan. We may find, for example, that the transition from the interactive discussion guide to the physical exam is unnatural, and we will adapt the tool to integrate more seamlessly into the clinical visit. PCPs will opine about the receipt and usefulness of the automatically generated survivorship care plan. If, for example, PCPs find the survivorship care plan too long, we may abbreviate the content. Responsiveness to stakeholder feedback will improve the intervention for future users.

This study will also provide critical baseline data to characterize this vulnerable population. Currently, little is known about the prevalence of late effects and the unmet late effect management needs among head and neck cancer survivors. The presence and management of comorbid conditions have also not been well characterized. Using the data from this feasibility study, a future trial can help to target the most salient needs in this vulnerable population.

HN-STAR must have the flexibility to function well in multiple environments in order to be feasible in a clinical trial. The two sites (MSK and HH) enable the assessment of differences with and without direct EMR integration of HN-STAR, informing the scalability of this intervention. If we find, for example, that the NPs in the two clinics provide conflicting feedback about how to adapt the interactive discussion guide for use in the visit, we may need to build more flexibility into HN-STAR so that it can be used differently in different settings.

Findings from this study will inform a multicenter randomized trial of HN-STAR, aimed at standardizing and streamlining the delivery of evidence-guided comprehensive care for head and neck cancer survivors. Ultimately, if found effective, the modular structure of HN-STAR could permit its expansion to survivors of other complex cancers.

### Trial status

The trial has received IRB approval and began enrollment in February 2016.
